# Is MYND Domain-Mediated Assembly of SMYD3 Complexes Involved in Calcium Dependent Signaling?

**DOI:** 10.3389/fmolb.2019.00121

**Published:** 2019-11-01

**Authors:** Yingxue Zhang, Chunying Li, Zhe Yang

**Affiliations:** ^1^Department of Biochemistry, Microbiology, and Immunology, Wayne State University School of Medicine, Detroit, MI, United States; ^2^Center for Molecular and Translational Medicine, Georgia State University, Atlanta, GA, United States

**Keywords:** SMYD3, MYND, PXLXP motif, signal transduction, PLCB3, protein complexes

## Abstract

Macromolecular complexes are essential to intracellular signal transduction by creating signaling niches and enabling a chain of reactions that transmit external signals into various cellular responses. Analysis of SMYD3 interactome indicates this protein lysine methyltransferase might be involved in calcium dependent signaling pathways through forming complexes with the phospholipase PLCB3, calcium/calmodulin dependent kinase CAMK2B, or calcineurin inhibitor RCAN3. SMYD3 is well-known as a histone H3K4 methyltransferase involved in epigenetic transcriptional regulation; however, any roles SMYD3 may play in signaling transduction remain unknown. KEGG pathway enrichment analysis reveals the SMYD3 interacting proteins are overrepresented in several signaling pathways such as estrogen signaling pathway, NOD-like receptor signaling pathway, and WNT signaling pathway. Sequence motif scanning reveals a significant enrichment of PXLXP motif in SMYD3 interacting proteins. The MYND domain of SMYD3 is known to bind to the PXLXP motif. The enrichment of the PXLXP motif suggests that the MYND domain is likely to be a key interaction module that mediates formation of some SMYD3 complexes. The presence of the PXLXP motifs in PLCB3 and CAMK2B indicates the potential role of the MYND domain in mediating complex formation in signaling. The structural basis of SMYD3 MYND domain-mediated interactions is unknown. The only available MYND-peptide complex structure suggests the MYND domain-mediated interaction is likely transient and dynamic. The transient nature will make this domain well-suited to mediate signaling transduction processes where it may allow rapid responses to cellular perturbations and changes in environment.

## Introduction

SMYD3 belongs to a special class of protein lysine methyltransferases that is classified by the presence of SET and MYND domains (Spellmon et al., 2015). SET domain is an evolutionarily conserved domain responsible for protein lysine methylation (Tian et al., 2013). MYND domain is a zinc finger motif involved in protein-protein interaction with transcriptional co-repressor complexes (Liu et al., 2007). SMYD3 is a ubiquitously expressed protein with the highest expression in brain and thyroid tissues (Uhlen et al., 2015). In cells, SMYD3 is found in both the nucleus and cytoplasm (Thul et al., 2017). Current research of SMYD3 focuses primarily on its roles in tumor cell growth and muscle development. SMYD3 expression is abnormally elevated in over 15 types of cancers (Spellmon et al., 2017). Overexpression of SMYD3 is associated with tumor cell growth (Hamamoto et al., 2004). While SMYD3 is required for the development of cardiac and skeletal muscles in zebrafish (Fujii et al., 2011), knockout of SMYD3 in mice shows no significant muscle phenotypes (Bagislar et al., 2016). However, in mice SMYD3 mediates the recruitment of transcriptional cofactors at the myostatin and c-Met genes and regulates skeletal muscle atrophy (Proserpio et al., 2013). Current understanding of the molecular mechanisms by which SMYD3 exerts its functions is largely limited to its roles in epigenetic control of gene expression. SMYD3 can upregulate expression of a myriad of oncogenes including c-MYC, STAT3, and matrix metalloproteinase-9 (Cock-Rada et al., 2012; Liu et al., 2015; Sarris et al., 2016). In almost all cases binding of SMYD3 to the promoter region of the target genes is associated with both H3K4 trimethylation and gene activation (Kim et al., 2009; Cock-Rada et al., 2012; Liu et al., 2012; Luo et al., 2014). However, the *in vitro* methyltransferase activity of SMYD3 toward H3K4 is much lower compared to other targets such as histone H4K5 (Van Aller et al., 2012). This has raised a question of whether H3K4 is a true *in vivo* epigenetic target (Van Aller et al., 2012; Mazur et al., 2014). SMYD3 also methylates non-histone proteins. Methylation of MAP3K2, a mitogen-activated protein kinase, links SMYD3 to the MAPK signaling pathway and Ras-driven cancer (Mazur et al., 2014). Methylation of vascular endothelial growth factor receptor-1 (VEGFR1) regulates VEGFR1 kinase activity potentially affecting VEGFR1-dependent angiogenesis (Kunizaki et al., 2007). While these studies have established the methyltransferase activity of SMYD3 being essential for its function, the catalytic-independent roles have been uncovered in several cases. Overexpression of a methylation-defective mutant of SMYD3 transactivated the promoter of MYC gene to a similar degree as that observed with the wild type protein (Sarris et al., 2016). SMYD3-mediated EMT (epithelial mesenchymal transition) gene regulation is also independent of SMYD3 methylation activity (Fenizia et al., 2019). Both the wild type and inactive SMYD3 mutant promoted upregulation of the mesenchymal genes N-cadherin and Vimentin and equally reduced E-cadherin and Occludin protein levels. Although the precise mechanism remains largely unknown, the protein-protein interaction events have been proposed to contribute to the catalytic-independent function of SMYD3 (Sarris et al., 2016; Fenizia et al., 2019).

### Involvement of SMYD3 in Signaling Pathways

#### SMYD3 Interacts With Proteins Involved in Signaling

**Figure 1:**
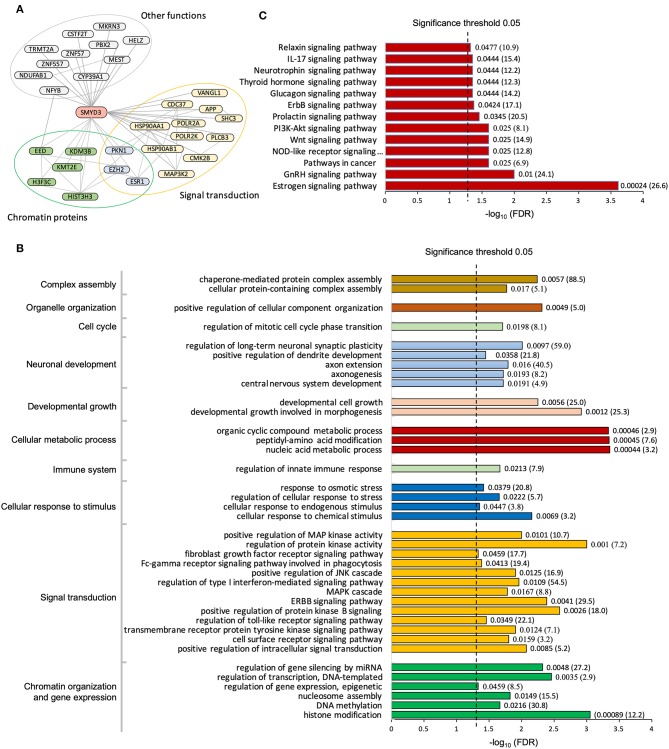
Pathway enrichment analysis of SMYD3 interactome. **(A)** SMYD3 interactors are enriched in two major functional groups: chromatin organization and signal transduction. Lines are drawn from SMYD3 to its interactors and between SMYD3 interactors if their interactions are documented in STRING with the interaction score larger than 0.3. A selected list of enriched GO biological processes **(B)** and KEGG pathways **(C)**. Numbers at the right end of the bars indicate the false discovery rate (FDR) and fold enrichment in parenthesis.

To date, a total of 43 human proteins were found to interact with SMYD3 (Stark et al., 2006; Fahey et al., 2011; Lopez et al., 2015; Szklarczyk et al., 2015). Among them 30 proteins were considered as high-confidence interactors based upon a reliability score for interaction (see Methods). We found these high-confidence interactors are enriched in two major functional groups, chromatin proteins that are involved in epigenetic transcriptional regulation and signaling molecules that are related to calcium dependent signaling (Figure 1A). Interacting with the phospholipase PLCB3 may link SMYD3 to calcium-dependent lipid signaling pathways. PLCB3 belongs to the phospholipase C (PLC) subtype that plays an important role in many signal transduction processes (Cocco et al., 2015). Many extracellular signals including many hormones, neurotransmitters, and growth factors converge their physiological action on PLC enzymes which subsequently propagate the signals leading to many cellular responses. PLC enzymes propagate extracellular signals by catalyzing the hydrolysis of the membrane bound phosphatidylinositol 4,5-bisphosphate (PIP2) into the second messengers inositol 1,4,5-trisphosphate (IP3) and diacylglycerol (DAG). IP3 and DAG then further propagate the signals by inducing the calcium release from endoplasmic reticulum and activating protein kinase C (PKC), respectively. Calcium is an essential downstream effector in PLC signal transduction pathways required for activation of many protein kinases including PKC and calcium/calmodulin dependent protein kinases (CAMKs). Therefore, interacting with CAMK2B further suggests SMYD3 might be involved in calcium-dependent signaling pathways. CAMK2B belongs to a family of serine/threonine specific kinases whose activation is initially dependent on the binding of calcium/calmodulin (Swulius and Waxham, 2008). CAMKs play an integral role in translating intracellular calcium signals to a variety of downstream targets. Upon activation, CAMK2B has been shown to phosphorylate a range of targets involved in learning, memory, synaptic plasticity, and long-term potentiation (Lisman et al., 2012; Zalcman et al., 2018).

Interacting with RCAN3 indicates SMYD3 might modulate additional aspects of calcium-dependent signaling. RCAN3 belongs to a family of small evolutionarily conserved proteins that can directly bind and inhibit calcineurin (Mulero et al., 2007). The inhibition regulates calcineurin-dependent signaling events in which calcineurin is activated by calcium and calmodulin. SMYD3 could thus indirectly affect calcineurin activation and function by binding to RCAN3. The best known function of calcineurin is to control the transcription of genes involved in cardiac hypertrophy development and T cell activation by dephosphorylating and activating nuclear factor of activated T cells (NFAT) (Molkentin, 2004; Hogan, 2017).

#### Pathway Analysis of SMYD3 Interactome

**Figure 2:**
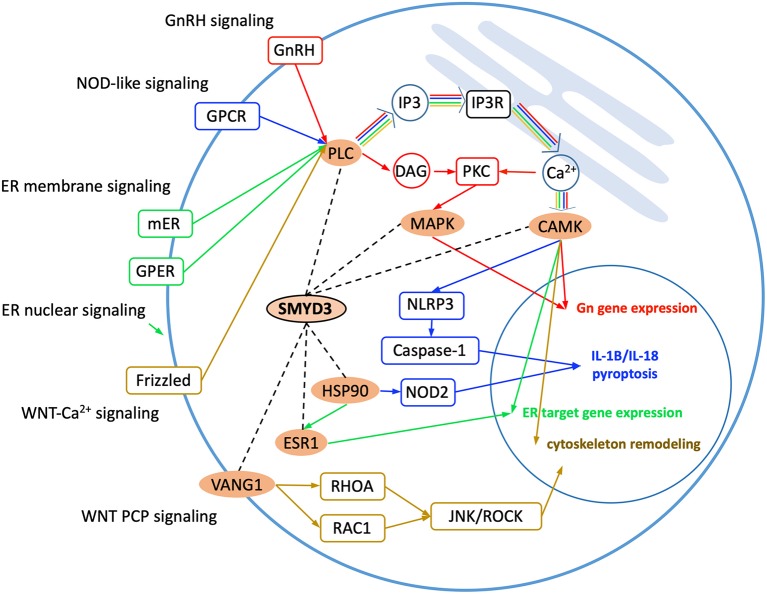
Enriched KEGG signaling pathways. SMYD3 interacting proteins are highlighted in orange. Dashed lines indicate the interactions between SMYD3 and its interactors. Arrow lines indicate signaling pathways with GnRH signaling, NOD-like signaling, ER signaling, and WNT signaling colored in red, blue, green, and yellow, respectively.

Gene Ontology analysis reveals the SMYD3 interacting proteins are enriched in positive regulation of intracellular signal transduction, cell surface receptor signaling, cellular response to chemical stimulus, and regulation of MAPK cascade (Figure 1B). KEGG (Kyoto Encyclopedia of Genes and Genome) pathway enrichment analysis also reveals these proteins are overrepresented in several signaling pathways including estrogen signaling pathway, GnRH signaling pathway, NOD-like receptor signaling pathway, and WNT signaling pathway (Figure 1C). In estrogen signaling, SMYD3 may interact with the proteins in both nuclear-initiated and membrane-initiated signaling pathways (Figure 2). In the nuclear pathway, SMYD3 may interact with estrogen receptor α (ERα) and the heat shock protein 90 (HSP90). The interaction between SMYD3 and the ligand binding domain of ERα is required for recruitment of SMYD3 to the proximal promoter regions of ER target genes (Kim et al., 2009). The recruited SMYD3 is responsible for the accumulation of di- and trimethylation of H3K4 and subsequent ER-regulated gene transcription. The role of its interaction with HSP90 is unknown. HSP90-based chaperone complex is thought to repress the ER transcriptional regulatory activities while maintaining the receptor in a conformation that is competent for high-affinity steroid binding (Knoblauch and Garabedian, 1999). However, HSP90 can enhance SMYD3 methyltransferase activity suggesting HSP90 could also positively regulate ER transcriptional activity through SMYD3 (Brown et al., 2015). In the membrane pathway, estrogen can exert its action through a subpopulation of ER at the plasma membrane (mER) or G-protein coupled E2 receptors (GPERs) (Song, 2007). SMYD3 may regulate mER signaling through binding to PLC. The PLC-mediated intracellular calcium release is central to the mechanism of mER signaling (Chaban et al., 2004). The released calcium can affect various downstream signaling pathways including calcium, cAMP and PI3K-AKT pathways and lead to activation of CREB family transcription factors and various ER cellular responses (Manavathi and Kumar, 2006).

Also through binding to the PLC SMYD3 may be involved in GnRH signaling and NOD-like receptor signaling (Figure 2). In GnRH signaling pathway, gonadotropin-releasing hormone (GnRH) acts upon its receptor in the anterior pituitary to regulate the production of gonadotropins (Perrett and McArdle, 2013). PLC is responsible for transducing the GnRH signal, which activates calcium signaling pathway, protein kinase C (PKC) pathway, and MAPK signaling pathway (Call and Wolfe, 1999). SMYD3 may regulate CAMK activity in calcium signaling and methylate MAP3K2 in MAPK signaling. In NOD-like receptor signaling pathway, PLCB-mediated calcium signaling affects NLRP3 inflammasome assembly and activation (Murakami et al., 2012). The activated NLRP3 induces caspase-1 activation, which then regulates maturation of the pro-inflammatory cytokines IL-1B and IL-18 and drives pyroptosis (He et al., 2016). SMYD3 may affect NOD2 signaling also through binding to HSP90. The proper functioning of NLR proteins (NOD2) is dependent on HSP90. It has been shown that NOD2 interacts with HSP90 via both CARD domains; the interaction enhances NOD2 stability (Boyle et al., 2014).

SMYD3 interacts with the proteins that play roles at several points in WNT signaling (Figure 2). WNT signaling is required for basic developmental processes such as cell-fate specification, progenitor-cell proliferation and the control of asymmetric cell division, in many different species and organs (Nayak et al., 2016). There are at least three different WNT pathways: the canonical pathway, the planar cell polarity (PCP) pathway and the WNT-Ca^2+^ pathway. SMYD3 interacts with the proteins involved in WNT-Ca^2+^ signaling and PCP signaling. WNT-Ca^2+^ signaling is mediated through G proteins and phospholipases and leads to transient increases in cytoplasmic free calcium that subsequently activates the kinase PKC and CAMK2 and the phosphatase calcineurin (Kuhl et al., 2000). In this signaling, SMYD3 may interact with PLCB, CAMK2, and the calcineurin inhibitor RCAN3. SMYD3 may regulate PCP signaling through binding to VANGL1. Van Gogh-like (VANGL) proteins are core components of the planar cell polarity pathway that controls epithelial polarity and cell migration (Mentink et al., 2018). PCP signaling leads to the activation of the small GTPases RHOA and RAC1, which activate the stress kinase JNK and ROCK and lead to remodeling of the cytoskeleton and changes in cell adhesion and motility (Yang and Mlodzik, 2015).

#### Functional Associations Between SMYD3 and Its Interactors

Appreciation of SMYD3 involvement in signaling would broaden our view of the role of SMYD3 in cancer beyond epigenetic regulation. The diversity of cellular signaling exploited by cancer is immense. Oncogenic mutations have been found in numerous signaling pathways including WNT signaling, calcium signaling, and MAPK signaling cascade (Sever and Brugge, 2015). In each case, the balance of signaling is disrupted by the mutations, which is not subject to the normal control mechanisms. Disrupted cell signaling allows cells to overproliferate and causes changes in the tumor microenvironment, angiogenesis, inflammation, and cell metabolism, and consequently promotes cancer progression and metastasis and evades apoptosis and immune destruction. The interaction of SMYD3 with proteins involved in these signaling pathways would provide additional mechanistic links between SMYD3 overexpression and cancers.

The available data also emphasize potential functional associations between SMYD3 and its interactors in normal physiology in addition to cancer. SMYD3 is a ubiquitously expressed protein with the highest expression levels in brain, thyroid, and reproductive organs (Uhlen et al., 2015). However, the precise roles of SMYD3 in these tissues remain completely unknown. The interaction with GnRH signaling could suggest a possible functional integration of SMYD3 in brain and testis. GnRH signaling is the primary regulator of mammalian reproductive function in both males and females controlling gametogenesis and steroidogenesis (Perrett and McArdle, 2013). The interaction with this signaling pathway would suggest that SMYD3 might be involved in the endocrine control of spermatogenesis by regulating the neuroendocrine activity along the hypothalamic-pituitary-testicular axis.

There might be potential functional connections between SMYD3 and CAMK2B in muscle atrophy. SMYD3 regulates skeletal muscle atrophy through the recruitment of transcriptional cofactors to the myostatin and c-Met genes (Proserpio et al., 2013). Reducing SMYD3 decreased myostatin and c-Met transcription, which prevented muscle loss and protected from glucocorticoid-induced myotube atrophy. CAMK2B is also linked to muscle atrophy. CAMK2B was identified as a downstream target of p38α MAPK and positively regulated muscle atrophy (Clarke et al., 2007). The pharmacological inhibition of CAMK2B activity suppressed denervation-induced muscle atrophy. SMYD3 could contribute to p38α MAPK-mediated muscle atrophy through binding to CAMK2B. CAMK2B was found to regulate MuRF1 transcription (Clarke et al., 2007). MuRF1 is an E3 ubiquitin ligase that degrades myosin heavy chain causing the breakdown of skeletal muscle under atrophy conditions (Yuasa et al., 2018). MuRF1 has been shown to be upregulated during both denervation and glucocorticoid treatment (Furlow et al., 2013). It is possible that SMYD3 could play multiple transcriptional regulatory roles in glucocorticoid-induced myotube atrophy. It is not only involved in epigenetic regulation of myostatin and c-Met genes but also could affect CAMK2B-dependent MuRF1 gene expression through binding to CAMK2B.

The interaction with NOD-like receptor signaling or PLCB3 might link SMYD3 to inflammation and the immune response. NOD-like receptors are specific families of pattern recognition receptors responsible for detecting various pathogens and generating innate immune responses (Mogensen, 2009). PLCB3 is required for FceRI-mediated mast cell activation (Xiao et al., 2011). Loss of PLCB3 leads to defective mast cell-dependent immune responses. PLCB3 also regulates the cystic fibrosis (CF) inflammatory response by amplifying the expression and release of IL-8 and recruiting neutrophils to CF airways (Bezzerri et al., 2011). SMYD3 has been shown to play a role in regulatory T cell development. SMYD3 regulates Foxp3 expression via its histone H3K4 methyltransferase activity (Nagata et al., 2015). This epigenetic control is an important mechanism in regulating TGFβ-dependent regulatory T cell formation. SMYD3 abrogation affected regulatory T cell formation while allowing dysregulated interleukin-17 production. The interaction with NOD-like receptor signaling and PLCB3 could suggest that SMYD3 might also be involved in innate immune response in addition to regulation of adaptive immunity.

### Structural Basis of the Formation of SMYD3 Complexes

#### Structures of SMYD3 and Its Substrate Complex

**Figure 3:**
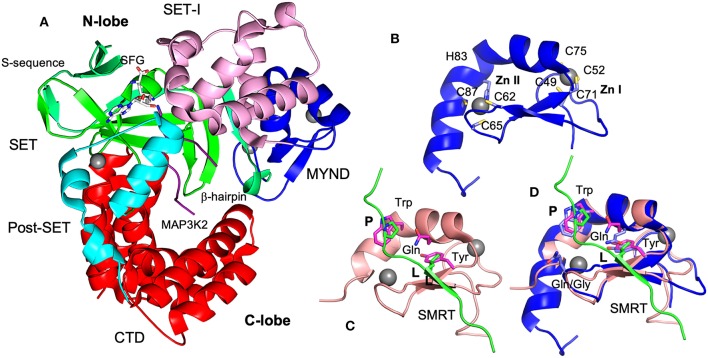
Structures of SMYD3 and MYND domains. **(A)** Ribbon diagram of SMYD3 structure (PDB code: 3PDN). The S-sequence, MYND, SET-I, core SET, post-SET, and CTD are depicted in light green, blue, pink, green, cyan, and red, respectively. The cofactor analog sinefungin (SFG) is represented by balls and sticks. MAP3K2 peptide (PDB code: 5EX0) is shown as a purple coil. **(B)** Structure of SMYD3 MYND domain. MYND is represented by ribbon. Zinc atoms are shown as spheres. Zinc chelating residues are depicted by sticks. **(C)** Structure of ETO MYND domain in a complex with a SMRT peptide (PDB code: 2ODD). MYND and the peptide are colored in pink and green, respectively. MYND residues involved in peptide binding are depicted by sticks and colored in magenta. The residues leucine (L) and first proline (P) in the peptide motif are represented by sticks and colored in green. **(D)** Structural superposition of the MYND domains of SMYD3 and ETO. Residues are colored according to the schemes in **(B,C)**. The overall root mean square difference (RMSD) between the two structures is 0.86 Å.

Like other SMYD proteins (SMYD1 and 2), SMYD3 structure is bilobal (Figure 3A) (Sirinupong et al., 2010, 2011; Jiang et al., 2011; Spellmon et al., 2015). The N-terminal lobe contains four domains: SET, MYND, SET-I, and post-SET. The C-terminal lobe contains a tetratricopeptide repeat (TPR)-like domain that is organized into seven up-down helices. There are at least three scenarios in that SMYD3 can form complex with other proteins. The SET domain is required for the formation of enzyme-substrate complexes (Spellmon et al., 2015). The MYND domain is a putative protein-protein interaction module that interacts with a proline-rich sequence (Liu et al., 2007). The TPR-like CTD domain has been shown to interact with HSP90 but the exact nature of such an interaction is unknown (Brown et al., 2015).

The structure of SMYD3 in complex with a MAP3K2 peptide provided the structural basis for SMYD3-substrate complex formation (Fu et al., 2016). The MAP3K2 peptide was found to bind at the bottom of a deep cleft formed between the N- and C-lobes (Figure 3A). Similar to the SMYD2-substrate complexes (Ferguson et al., 2011; Wang et al., 2011; Jiang et al., 2014), the bound peptide adopts a U shaped conformation. The base of the U shape is sandwiched between a β-hairpin from the SET domain and a loop preceding the post-SET domain. The descending arm of the U shape is bound by the SET-I domain, while the ascending arm interacts with the CTD. The target lysine is located within the U base. The side chain of the target lysine is recognized by a long narrow channel through which it is deposited into the active site. The structural basis of SMYD3-non-substrate complexes remains unknown.

#### MYND Domain-Mediated Protein Interaction

The MYND is likely an important module that contributes to the assembly of several SMYD3 complexes. MYND domain is present in about 90 human proteins (Hunter et al., 2009). The role of MYND is largely unexplored and limited to protein interaction and recruitment of transcriptional co-repressors (Liu et al., 2007). In the SMYD protein family, SMYD1 is known to interact with skNAC through its MYND domain (Sims et al., 2002). Cysteine to serine mutations in the first or second zinc fingers of the MYND domain abolished the ability of SMYD1 to bind skNAC in immunoprecipitation assays. The interaction of SMYD2 with EPB41L3 is also mediated by the MYND domain (Abu-Farha et al., 2008). The wild type but not the MYND-deleted form of SMYD2 was found to co-immunoprecipitate with EPB41L3. The MYND domain of SMYD3 is also required for its binding to the co-repressor N-CoR (Foreman et al., 2011). N-CoR co-immunoprecipitated with wild type SMYD3 but not with the MYND domain point mutant C49S. MYND domain appears to prefer interacting with a proline-rich sequence. The MYND of BS69 was found to bind to a PXLXP motif (Ansieau and Leutz, 2002). A leucine to alanine mutation in the PXLXP motif abolished the interaction. ETO and DEAF-1 MYND domains were found to interact with PPPLI motif (Liu et al., 2007; Kateb et al., 2013). Disrupting the interaction between the ETO MYND domain and the SMRT PPPLI motif attenuated AML1/ETO effects on proliferation, differentiation, and gene expression (Liu et al., 2007). The MYND domains of SMYD proteins are likely to bind to the PXLXP motif (Sims et al., 2002; Abu-Farha et al., 2008; Foreman et al., 2011). Co-immunoprecipitation experiments performed in transiently transfected 293T cells identified the PPLIP motif in skNAC as an important SMYD1 interaction motif as the substitution of the leucine to alanine failed to associate with SMYD1 (Sims et al., 2002).

MYND domain adopts a conserved β-β-α topology in which two anti-parallel β-strands and one small kinked α-helix organize around two zinc atoms. The zinc atoms are chelated by seven cysteine residues and one histidine in a C4-C2HC arrangement (Figure 3B). The solution structure of ETO in complex with a PPPLI sequence from the co-repressor SMRT revealed the peptide binding mode and residues responsible for peptide recognition (Liu et al., 2007). The bound peptide adopts a stretched conformation (Figure 3C). It is connected to the MYND domain by forming a β strand that pairs with the β strands of the MYND domain. The residue leucine (L) in the peptide binds at a shallow surface pocket containing the residues tyrosine and glutamine. The first proline (P) is packed against by a tryptophan residue. There are no significant specific interactions between the rest positions of the peptide and MYND domain.

MYND domain-based interactions show relatively low binding affinities with K_D_ in the millimolar range (Kateb et al., 2013). The low binding affinities are consistent with the structural finding that ETO and SMRT bind with much smaller interface areas (~400 Å^2^). The weak interaction suggests the MYND-mediated interactions are transient in nature. Transient interactions usually show a fast bound-unbound equilibrium and can readily switch between different binding partners (Perkins et al., 2010). However, in some cases effective MYND interaction requires additional contacts between the other regions of proteins. The N-terminal residues located in the S-sequence of SMYD1 are required for efficient interaction with the PXLXP motif in skNAC (Sims et al., 2002). A set of positively charged residues located at the C-terminus of BS69 MYND domain is crucial for the interaction with PXLXP ligands (Kateb et al., 2013).

The structural basis of SMYD MYND domains-mediated interactions is unknown. Based on the structural superposition (Figure 3D), the peptide binding mode in SMYD proteins could be largely similar to that of ETO. The leucine and first proline of the peptide could be similarly bound since the residues responsible for binding to these two positions are conserved and well-structurally aligned between SMYD proteins and ETO. In SMYD3, three conserved residues Trp80, Gln76, and Tyr70 may contribute to the binding of the PXLXP motif. The tryptophan residue may pack against the first proline, and the glutamine and tyrosine residues may form a hydrophobic pocket for the leucine to bind. However, due to a sequence variation between the third and fourth zinc-chelating cysteines there are structural differences in the peptide binding sites. Such differences might be associated with the different binding specificities. In ETO a glycine residue between the third and fourth zinc-chelating cysteines is replaced by a glutamine residue in SMYD proteins. As a result, their structures differ in the peptide binding pocket that binds to the peptide region between the leucine and first proline. Since the PXLXP motif bound by SMYD proteins is one residue shorter than the ETO-bound PPPLI sequence at this region, this pocket may contribute to binding specificity. The structural difference in this pocket may only allow the corresponding peptide motif to be accommodated. To date there are no structures available for PXLXP-complexed MYND domains and the exact binding mode for this motif is unknown.

In contrast to binding specificity, MYND domains also show promiscuous binding. Many MYND domains are capable of binding to multiple targets, and single peptide motifs can be recognized by different MYND domains. The MYND domain of BS69 is able to bind to MGA, E1A, and EBNA2 proteins (Ansieau and Leutz, 2002), while binding to SMRT and N-CoR appears to be a common feature of many MYND domains including those of ETO, RACK7, and DEAF-1 (Liu et al., 2007; Kateb et al., 2013). Additionally, the promiscuous binding can be due to the coexistence of several proline-rich motifs in one target. N-CoR contains the overlapping PPPLI and PLXLP motifs and can then be bound by different classes of MYND domains (TRPPPPLIPSSK). The structure of ETO-SMRT complex has revealed limited specific interactions between the MYND domain and peptide (Figure 3C). The less constrained structural features in the peptide binding site would allow for a broader binding specificity providing some structural explanation of promiscuous MYND binding.

#### Enrichment of PXLXP Sequences in SMYD3 Interactors

**Figure 4:**
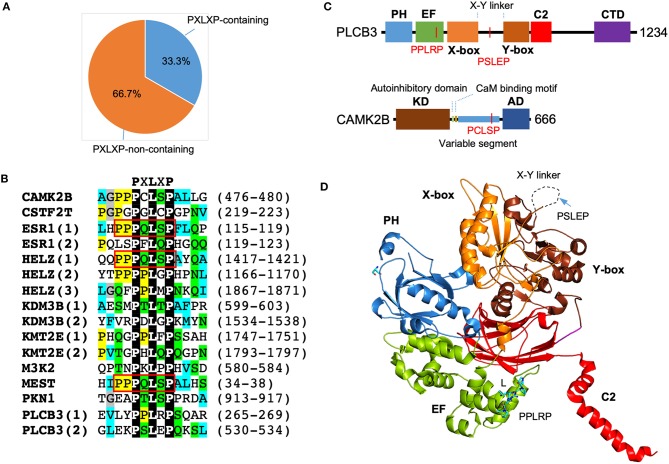
Enrichment of PXLXP motif in SMYD3 interacting proteins. **(A)** The percentage of PXLXP containing proteins in SMYD3 interactors. **(B)** Sequence alignment of SMYD3 interacting proteins at PXLXP motif. Conserved motif-residues are shown as white on black. Other residues are colored according to ClustalX grouping scheme (Larkin et al., 2007): proline (yellow), glycine (gray), small or hydrophobic (C, A, V, L, I, M, F, W) (cyan), hydroxyl or amine (S, T, N, Q) (green), charged (D, E, R, K), and histidine or tyrosine (H, Y). Residues are colored only if the percentage of residues from a group is larger than 25%. Numbering at the right end of the sequences indicates the start and end of PXLXP motif. Red boxes indicate identical PQLSP motif. **(C)** Domain structure of PLCB3 and CAMK2B. In PLCB3, PH, pleckstrin homology domain; EF, EF-hands domain; X-box, catalytic X domain; Y-box, catalytic Y domain; C2, C2 domain; CTD, C-terminal domain. In CAMK2B, KD, kinase domain; AD, self-association domain; CaM, calmodulin. Red lines indicate the positions of PXLXP motifs. **(D)** Ribbon diagram of PLCB3 structure (PDB code: 3OHM). PLCB3 domains are colored according to the scheme in **(C)**. Dash line indicates disordered X-Y linker. PPLRP motif is depicted by sticks. PSLEP motif is indicated by an arrow.

Motif scanning using ScanProsite (de Castro et al., 2006) reveals a significant enrichment of PXLXP motif in SMYD3 interacting proteins. A total of 16 matches was found in 10 proteins including PLCB3, CAMK2B, PKN1, ESR1, HELZ, KDM3B, KMT2E, CSTF2T, MEST, and MAP3K2. In other words, 33.3% of SMYD3 interacting proteins contain 1 or more PXLXP motifs, and the match percentage is 53.3% (Figure 4A). The chance for a random match for this motif is 10.3% (Nicodeme, 2001). This indicates more than 5.1-fold enrichment of PXLXP motif in SMYD3 interacting proteins. Sequence alignment of all 16 matches reveals a specific PQLSP motif occurs three times each in ESR1, HELZ, and MEST (Figure 4B). This occurrence represents more than 330-fold enrichment. Of note, the flanking sequences of this motif are also conserved containing additional proline residues (PPPQLSPF).

The significant enrichment of PXLXP motif in SMYD3 interacting proteins suggests that the MYND domain is likely to be a key interaction module that mediates the formation of some SMYD3 complexes. The presence of PXLXP motifs in PLCB3 and CAMK2B indicates the potential role of the MYND domain in mediating complex formation in signaling including the aforementioned estrogen signaling pathway, GnRH signaling pathway, NOD-like receptor signaling pathway, and WNT signaling pathway. In PLCB3 there are two PXLXP motifs, PPLRP at residues 265–269 and PSLEP at residues 530–534 (Figure 4B). The former motif is located within the EF-hand repeats, and the latter motif is located at the X-Y linker connecting the X and Y halves of the triose phosphate isomerase (TIM)-like barrel domain (Figure 4C). As shown by the crystal structure of PLCB3, the latter motif appears to be more accessible for binding than the former motif (Figure 4D). The central leucine (L) in the former motif is buried inside the EF-hand domain and involved in intramolecular interactions. With its current conformation it is essentially impossible for this motif to interact with MYND domains unless there is a significant conformational change. In contrast, the X-Y linker where the latter motif is located is unstructured and likely located on the top of the protein surface. With this flexibility and location, the latter motif should be more accessible for binding with minimum steric hindrance. Additionally, the latter motif appears to be electrostatically more compatible with the MYND domain of SMYD3. The latter motif, PSLEP is negatively charged, whereas the former motif, PPLRP positively charged. The MYND domains of SMYD proteins have a highly positively charged surface and likely prefer binding to a negatively charged peptide (Spellmon et al., 2015). If the MYND domain binds to the latter motif, this binding could potentially regulate PLCB3 phospholipase activity and subsequently signal transduction processes, since the X-Y linker has been shown to play an autoinhibitory role (Hicks et al., 2008).

CAMK2B has only one PXLXP motif, PCLSP at residues 476–480. This motif is located at the variable segment of CAMK2B (Figure 4C). The variable segment of CAMK2B plays a role in targeting the holoenzyme CAMK2A/2B to F-actin cytoskeleton and localizing the holoenzyme to dendritic spines (Shen et al., 1998). The targeting occurs at basal Ca^2+^ concentrations; as intracellular Ca^2+^ concentrations rise, the holoenzyme is released from the cytoskeleton and enters the cytosol (Swulius and Waxham, 2008). Binding to the cytoskeleton limits protein diffusion and has been thought as a mechanism to restrict the activation of the holoenzyme and subsequently the phosphorylation of its substrates (Swulius and Waxham, 2008). Since the PCLSP motif is located within the variable segment of CAMK2B, if this motif can be bound by the MYND domain of SMYD3, such binding could potentially regulate CAMK2B cytoskeletal targeting and the activation of the holoenzyme. The PCLSP motif may have a physiological role in signaling, as the serine residue in this motif is phosphorylated in the signaling pathways downstream of SDF-1/CXCR4 in breast cancer stem cells (Yi et al., 2014). However, the PCLSP motif is not present in mouse and rat proteins and also missing in other CAMK2 isoforms (UniProt Consortium, 2019). This unique feature might be relevant to the role of CAMK2B in human-specific synaptic plasticity and the functional differences between CAMK2A and CAMK2B in F-actin binding (Shen et al., 1998).

## Discussion

Analysis of SMYD3 interactome reveals SMYD3 might be involved in signal transduction pathways in addition to epigenetic gene regulation. Most enriched KEGG pathways are associated with the signaling processes in cancer. Among them, the WNT signaling has been shown to affect the maintenance of stemness and metastasis of cancer cells (Zhan et al., 2017), and the MAPK signaling can enhance cell proliferation and angiogenesis (Dhillon et al., 2007). This indicates that SMYD3 might adopt an integrated model that combines signaling and epigenetic pathways to contribute to tumor cell proliferation and growth. One notable interaction is with the phospholipase PLCB3. PLC enzymes represent convergence points for many signal transduction pathways. PLCB3 is known to regulate opioid-dependent signaling and is required for opioid-induced calcium release in the nervous system (Xie et al., 1999). PLCB3 also plays a role in *Pseudomonas aeruginosa*-induced intracellular calcium signaling and regulates the activation of PKC and the nuclear transcription factor NF-κB in human bronchial epithelial cells (Bezzerri et al., 2011). The interaction with PLCB3 would suggest that SMYD3 might be involved in PLCB3-dependent signaling pathways to regulate free calcium transients. One key question needed to be addressed is where the interaction between SMYD3 and PLCB3 occurs in cell. SMYD3 is present in both cytosol and nucleus, whereas PLCB3 is primarily a peripheral membrane protein but also found in nucleus (Thul et al., 2017). The translocation of PLCB3 from the nucleus to the plasma membrane is a controlled process that regulates its activation and subsequently IP3-induced calcium signaling in Jurkat T-cells (Xie et al., 1999). If the interaction occurs in the nucleus, SMYD3 could potentially affect the nuclear export of PLCB3. SMYD3 has been shown to regulate nucleo-cytoplasmic shuttling of Human T-lymphotropic Virus 1 (HTLV-1) Tax protein by direct interaction with Tax and tethering Tax in the nucleus of T cells (Alefantis et al., 2005). Therefore, knowledge of spatial interaction between SMYD3 and PLCB3 at the subcellular level would elucidate more detailed mechanistic insights into the role of SMYD3 in PLCB3-dependent signaling pathways in terms of regulation of PLCB3 nuclear export or its phospholipase activity at the plasma membrane.

The MYND domain might be a key complex-forming module in SMYD3 complexes. The PXLXP motif to which the MYND domain may bind is significantly enriched in SMYD3 interacting proteins. However, there is no significant difference in the enrichment between the interactors involved in signaling transduction pathways and epigenetic transcriptional regulation suggesting that the MYND domain does not have any particular binding preferences in either of these processes. No MYND domain has been directly linked to signaling, but some MYND domain-containing proteins such as BRAM1 as well as its *Caenorhabditis elegans* orthologs, Bra-1 and Bra-2, have been shown to participate in BMP and TGFβ signaling pathways (Kurozumi et al., 1998; Morita et al., 2001). For SMYD3, its MYND domain could be involved in PLCB3-mediated signaling pathways since PLCB3 contains the PXLXP motifs. The probable MYND binding motif in PLCB3, PSLEP is located within the X-Y linker, a sequence region highly variable across PLCB isozymes and also across species. The PSLEP motif is not present in other PLCB isozymes while it shows a modest degree of conservation across species (UniProt Consortium, 2019). The motif is conserved in most vertebrate animals including higher primates, horse, dog, sheep, and some rodents. The motif is not conserved in bird, fish, reptile, some rodents including mouse and rat, some lower primates such as galago, and all invertebrate animals. The modest conservation or divergence of the PSLEP motif might be related to the regulatory roles of the X-Y linkers. The very divergent primary sequences of the various X-Y linkers are likely the results of lineage- or species-specific evolution that creates distinct modes of autoregulation, therefore helping to respond to multiple extra- and intracellular inputs with appropriate enzymatic kinetics. The structural basis of SMYD3 MYND domain-mediated interactions is unknown. The only available MYND-peptide complex structure suggests the MYND domain-mediated interaction is likely transient and dynamic (Liu et al., 2007). Transient interactions are frequently involved in signaling that allow rapid responses to cellular perturbations and changes in environment (Stein et al., 2009; Perkins et al., 2010). The transient nature of MYND-domain mediated interactions would make this domain well-suited to mediate signaling transduction processes.

## Methods

### Selection of High-Confidence SMYD3 Interacting Proteins

SMYD3 interacting proteins were retrieved from four protein-protein interaction databases including GPS-Prot (Fahey et al., 2011), BioGRID (Stark et al., 2006), HitPredict (Lopez et al., 2015), and STRING (Szklarczyk et al., 2015). GPS-Prot archives 38 experimentally determined interactors from human, BioGRID 29, HitPredict 33, and STRING 15; a total of 43 unique interactions. The final list of proteins used in this study was generated by removing interactors with low-confidence reliability scores. We considered proteins as the low-confidence interactors if their interaction score is <0.3 in STRING or labeled as “low” in HitPredict. The final list contains 30 interactors.

### Pathway Enrichment Analysis

Pathway enrichment analysis of SMYD3 interactors was performed using STRING web-based analyzer (Szklarczyk et al., 2015). Pathway resources used are the Gene Ontology (GO) knowledgebase (The Gene Ontology Consortium, 2019) and Kyoto Encyclopedia of Genes and Genomes (KEGG) database (Kanehisa et al., 2019). All human proteins were used as the background population in the analysis. Evidence for enrichment in annotated pathways was evaluated using the Benjamini and Hochberg procedure that controls the false discovery rate (FDR) for multiple comparisons. An enrichment was considered as significant if the FDR is <0.05.

### PXLXP Motif Enrichment Analysis

The presence of PXLXP motif in SMYD3 interacting proteins was scanned using ScanProsite (de Castro et al., 2006). Enrichment analysis was performed by comparing the number of matches to the expected random matches of the motif in the background, i.e., ~100,000 sequences or 50,000,000 residues (Nicodeme, 2001). Fold enrichment was calculated as the ratio of the match percentage within our protein list to the percentage of the random matches against the background. The expected random matches are 10318 for PXLXP motif, and 30 for PQLSP motif (Nicodeme, 2001). Significance of enrichment was evaluated using the Benjamini and Hochberg procedure. FDR <0.05 was considered as significant.

## Data Availability Statement

All datasets generated for this study are included in the article/supplementary material.

## Author Contributions

ZY and YZ contributed conception and design of the study, performed the database and statistical analyses, and prepared the figures. ZY wrote the first draft of the manuscript. All authors contributed to manuscript revision, read, and approved the submitted version.

### Conflict of Interest

The authors declare that the research was conducted in the absence of any commercial or financial relationships that could be construed as a potential conflict of interest.
